# Crystal structure of 5-[(4-carb­oxy­benz­yl)­oxy]isophthalic acid

**DOI:** 10.1107/S2056989016011762

**Published:** 2016-07-29

**Authors:** Md. Serajul Haque Faizi, Musheer Ahmad, Akram Ali, Vadim A. Potaskalov

**Affiliations:** a Department of Chemistry, College of Science, Sultan Qaboos University, PO Box, 36 Al-Khod 123, Muscat, Sultanate of Oman; b Department of Applied Chemistry, Aligarh Muslim University, 202 002 UP, India; c National Taras Shevchenko University, Department of Chemistry, Volodymyrska str. 64, 01601 Kyiv, Ukraine; d Department of General and Inorganic Chemistry, National Technical University of Ukraine, Kyiv Polytechnic Institute, 37 Prospect Peremogy, 03056 Kiev, Ukraine

**Keywords:** crystal structure, tri­carb­oxy­lic acid, CIA, carboxyl­ates, coordination polymers, metal–organic frameworks, hydrogen bonding

## Abstract

The mol­ecular shape of the title compound, C_16_H_12_O_7_, is bent around the central CH_2_—O bond. The two benzene rings are almost perpendicular to one another, making a dihedral angle of 87.78 (7)°. In the crystal, each mol­ecule is linked to three others by three pairs of O—H⋯O hydrogen bonds, forming undulating sheets parallel to the *bc* plane and enclosing *R*_2_^2^(8) ring motifs. The sheets are linked by C—H⋯O hydrogen bonds and C—H⋯π inter­actions, forming a three-dimensional network.

## Abstract 2

The title compound, 5-[(4-carb­oxy­benz­yl)­oxy]isophthalic acid (CIA), is non-planar with the two benzene rings being almost perpendicular to one another, making a 87.78 (7)°. In the crystal, mol­ecules are linked by three pairs of O—H⋯O hydrogen bonds, forming undulating sheets parallel to the *bc* plane and enclosing 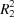
(8) ring motifs.

## Chemical context   

The design and synthesis of coordination polymers continues to attract inter­est due to their architectures as well as their potential applications (Erxleben, 2003 ▸). Recently, the rational design and synthesis of novel coordination polymers have attracted intense attention in the field of supra­molecular chemistry and crystal engineering (Zhang *et al.*, 2011 ▸). To date, large numbers of coordination architectures with inter­esting compositions and properties have been prepared using a wide variety of aromatic polycarboxyl­ate-based ligands (Cambridge Structural Database; Groom *et al.*, 2016 ▸). The title compound (CIA), a tri­carboxyl­ate ligand, has been shown to be a good candidate for the construction of coordination polymers (Ahmad *et al.*, 2012*a* ▸,*b* ▸). Tri­carboxyl­ate ligands have been used in the synthesis of metal-organic framework complexes (MOFs)because of their photoelectric properties and for their potential nitro­benzene sensing (Hou *et al.*, 2016 ▸). A Cd^II^ MOF based on CIA has been structurally and functionally characterized, and was shown to be an highly selective CH_2_Cl_2_ fluorescent sensor (Xia *et al.*, 2015 ▸). A series of one-, two- and three-dimensional coordination polymers based on CIA have been structurally characterized and shown to display photoluminescence (Liu *et al.*, 2012 ▸).

We have crystallized a reported polycarboxyl­ate containing the ligand, 5-[(4-carb­oxy­benz­yl)­oxy]isophthalic acid (CIA), which has the advantage of being flexible and has conformational freedom allowing it to conform to the coordination environment of transition metal ions. We report herein on the crystal structure of the title tri­carboxyl­ate ligand (CIA), synthesized by a reported procedure (Ahmad *et al.*, 2012*a* ▸,*b* ▸).
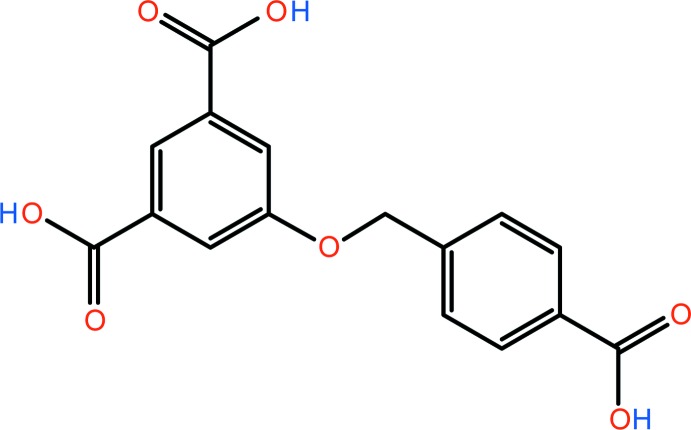


## Structural commentary   

**Figure 1:**
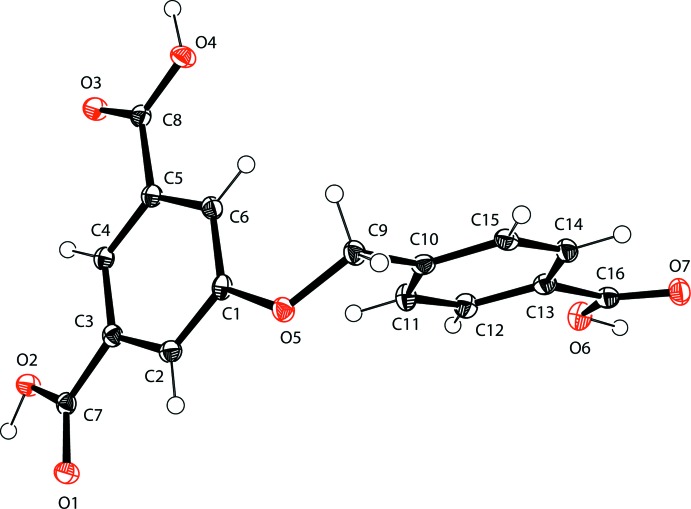
The mol­ecular structure of the title compound, with atom labelling. Displacement ellipsoids are drawn at the 40% probability level.

The mol­ecular structure of the title compound (CIA) is illus­trated in Fig. 1 ▸. The bond lengths and bond angles are normal and close to the values observed in related structures (Li & Ma 2011 ▸; He *et al.*, 2014 ▸). The mol­ecular shape of the title compound is bent around the central C9—O5 bond; the spacer ether group exhibits a C10—C9—O5—C1 torsion angle of −84.35 (19)°. The benzene rings, C1–C6 and C10–C15, are roughly perpendicular to each another, with a dihedral angle of 87.78 (7)°. The three O=C—O bond angles of the carb­oxy­lic acid groups are 123.17 (17), 123.62 (17), 123.74 (17) Å, respectively, for O1=C7—O2, O3=C8—O4 and O6=C16—O7. 

## Supra­molecular features   

**Figure 2:**
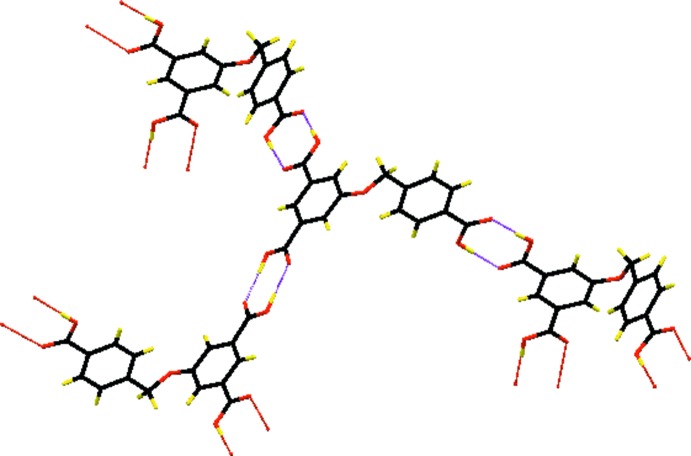
A partial view of the O—H⋯O hydrogen-bonding inter­actions between the donor and acceptor oxygen atoms of the carb­oxy­lic groups in the crystal of the title compound. The hydrogen bonds are shown as dashed lines (see Table 1 ▸ for details).

**Figure 3:**
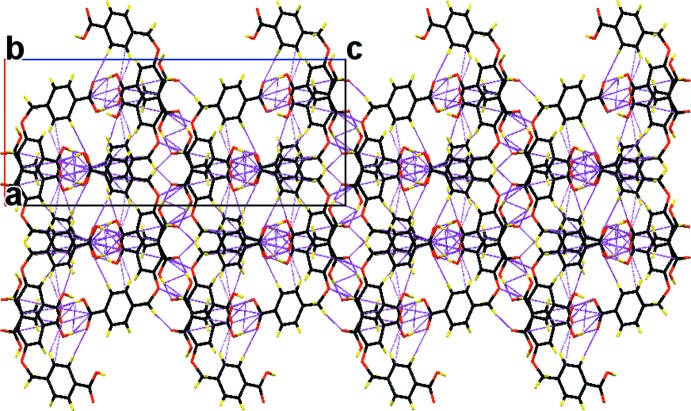
A view along the *b* axis of the crystal packing of the title compound. The O—H⋯O and C—H⋯O hydrogen bonds and C—H⋯π inter­actions are shown as dashed lines (see Table 1 ▸ for details). Fig 3 hard to make out, too many molecules shown

In the crystal, each mol­ecule is linked to three others by three pairs of O—H⋯O hydrogen bonds, forming undulating sheets parallel to the *bc* plane and enclosing 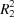
(8) ring motifs (Table 1 ▸ and Fig. 2 ▸). The sheets are linked by C—H⋯O hydrogen bonds and C—H⋯π inter­actions, forming a three-dimensional network (Table 1 ▸ and Fig. 3 ▸).

## Database survey   

A search of the Cambridge Structural Database (Version 5.37, update February 2016; Groom *et al.*, 2016 ▸) for the title compound gave 42 hits. The majority of these compounds are coordination polymers involving a secondary ligand. Ten structures concern coordination polymers of the title ligand itself. For example, *catena*-[μ_6_-5-(4-carb­oxy­benz­yloxy)isophthalato-(μ_2_-aqua)­barium(II)] where only two of the carb­oxy­lic acid groups of the CIA mol­ecule are deprotonated (BEDJOL; Li & Ma, 2012 ▸), and *catena*-[(bis­{μ_6_-5-[(4-carb­oxy­benz­yl)­oxy]isophthalato}(μ_2_-aqua))tricadmium trihydrate] (IZEBEV; Zhang *et al.*, 2011 ▸) where all three carb­oxy­lic acid groups of the CIA mol­ecule are deprotonated.

## Synthesis and crystallization   

The starting compound diethyl 5-(4-meth­oxy­carbonyl­benz­yloxy)isophthalate (DMBI) was prepared by the following procedure: 5-hy­droxy­isophthalic acid diethyl ester (2 g, 8.4 mmol) and dry K_2_CO_3_ (1.7g, 12.6 mmol) were mixed in dry aceto­nitrile (10 ml) and stirred for 30 min at 353 K. Then 4-bromo­methyl benzoic acid methyl ester (1.9 g, 8.40 mmol) was added and the resulting solution was refluxed for 24 h. The solution was pored into ice-cold water and the solid precipitate obtained was filtered and dried in air (yield: 2.8 g, 86%). The title compound (CIA) was prepared as follows: DMBI (2 g, 5.17 mmol) was hydrolyzed by refluxing it with 6*N* NaOH solution (20 ml) for 24 h. After cooling to 278 K, the resulting solution was acidified with 6*N* HCl solution to obtain a white precipitate. This was collected by filtration, washed thoroughly with water, and dried in air. The solid powder was dissolved in dimethyl formamide and needle-like crystals were obtained by slow diffusion of diethyl ether into the solution, after 2–3 days (yield: 1.3 g, 80%).

## Refinement   

Crystal data, data collection and structure refinement details are summarized in Table 2 ▸. The OH H atoms were located in a difference Fourier map and freely refined. The C-bound H atoms were positioned geometrically and refined using a riding model: C—H = 0.93-0.97 Å with *U*_iso_(H) = 1.2*U*_eq_(C).

## Supplementary Material

Crystal structure: contains datablock(s) I, global. DOI: 10.1107/S2056989016011762/su5300sup1.cif

Structure factors: contains datablock(s) I. DOI: 10.1107/S2056989016011762/su5300Isup2.hkl

Supporting information file. DOI: 10.1107/S2056989016011762/su5300Isup3.cml

CCDC reference: 1494816

Additional supporting information: crystallographic information; 3D view; checkCIF report

## Figures and Tables

**Table 1:** Hydrogen-bond geometry (Å, °) *Cg*2 is the centroid of the C10–C15 ring.

*D*—H⋯*A*	*D*—H	H⋯*A*	*D*⋯*A*	*D*—H⋯*A*
O2—H2*A*⋯O1^i^	0.95 (3)	1.70 (3)	2.6426 (19)	176 (2)
O4—H4*A*⋯O7^ii^	0.99 (3)	1.62 (3)	2.6086 (19)	175 (3)
O6—H6*A*⋯O3^iii^	0.93 (3)	1.72 (3)	2.6486 (19)	179 (4)
C9—H9*A*⋯O1^iv^	0.97	2.51	3.272 (2)	135
C11—H11*A*⋯O3^v^	0.93	2.45	3.170 (2)	135
C14—H14*A*⋯O4^vi^	0.93	2.56	3.245 (2)	131
C9—H9*B*⋯*Cg*2^vii^	0.97	2.97	3.779 (2)	141

Symmetry codes: (i) 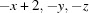; (ii) 
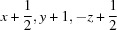
; (iii) 
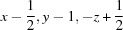
; (iv) 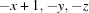; (v) 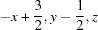; (vi) 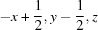; (vii) 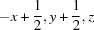.

**Table 2:** Experimental details

Crystal data	
Chemical formula	C_16_H_12_O_7_
*M*_r_	316.26
Crystal system, space group	Orthorhombic, *P* *b* *c* *a*
Temperature (K)	100
*a*, *b*, *c* (Å)	10.998 (2), 9.2760 (17), 25.661 (5)
*V* (Å^3^)	2618.0 (8)
*Z*	8
Radiation type	Mo *K*α
μ (mm^−1^)	0.13
Crystal size (mm)	0.27 × 0.21 × 0.16

Data collection	
Diffractometer	Bruker SMART APEX
Absorption correction	Multi-scan (*SADABS*; Bruker, 2003 ▸)
*T*_min_, *T*_max_	0.966, 0.980
No. of measured, independent and observed [*I* > 2σ(*I*)] reflections	12591, 2292, 1912
*R*_int_	0.043
(sin θ/λ)_max_ (Å^−1^)	0.595

Refinement	
*R*[*F* ^2^ > 2σ(*F* ^2^)], *wR*(*F* ^2^), *S*	0.040, 0.102, 1.07
No. of reflections	2292
No. of parameters	220
H-atom treatment	H atoms treated by a mixture of independent and constrained refinement
Δρ_max_, Δρ_min_ (e Å^−3^)	0.25, −0.24

Computer programs: *SMART* and *SAINT* ( Bruker, 2003 ▸), *SIR97* (Altomare *et al.*, 1999 ▸), *DIAMOND* (Brandenberg & Putz, 2006 ▸), *SHELXL97* (Sheldrick, 2008 ▸) and *PLATON* (Spek, 2009 ▸).

## supplementary crystallographic information

### Crystal data

**Table:**

C_16_H_12_O_7_	*F*(000) = 1312
*M**_r_* = 316.26	*D*_x_ = 1.605 Mg m^−^^3^
Orthorhombic, *P**b**c**a*	Mo *K*α radiation, λ = 0.71073 Å
Hall symbol: -P 2ac 2ab	Cell parameters from 999 reflections
*a* = 10.998 (2) Å	θ = 2.2–25.5°
*b* = 9.2760 (17) Å	µ = 0.13 mm^−^^1^
*c* = 25.661 (5) Å	*T* = 100 K
*V* = 2618.0 (8) Å^3^	Needle, colourless
*Z* = 8	0.27 × 0.21 × 0.16 mm

### Data collection

**Table:**

Bruker SMART APEX diffractometer	2292 independent reflections
Radiation source: fine-focus sealed tube	1912 reflections with *I* > 2σ(*I*)
Graphite monochromator	*R*_int_ = 0.043
/w–scans	θ_max_ = 25.0°, θ_min_ = 2.4°
Absorption correction: multi-scan (SADABS; Bruker, 2003)	*h* = −13→12
*T*_min_ = 0.966, *T*_max_ = 0.980	*k* = −11→10
12591 measured reflections	*l* = −30→19

### Refinement

**Table:**

Refinement on *F*^2^	Primary atom site location: structure-invariant direct methods
Least-squares matrix: full	Secondary atom site location: difference Fourier map
*R*[*F*^2^ > 2σ(*F*^2^)] = 0.040	Hydrogen site location: inferred from neighbouring sites
*wR*(*F*^2^) = 0.102	H atoms treated by a mixture of independent and constrained refinement
*S* = 1.07	*w* = 1/[σ^2^(*F*_o_^2^) + (0.0482*P*)^2^ + 1.0476*P*] where *P* = (*F*_o_^2^ + 2*F*_c_^2^)/3
2292 reflections	(Δ/σ)_max_ < 0.001
220 parameters	Δρ_max_ = 0.25 e Å^−^^3^
0 restraints	Δρ_min_ = −0.24 e Å^−^^3^

### Special details

**Table:**

Geometry. All esds (except the esd in the dihedral angle between two l.s. planes) are estimated using the full covariance matrix. The cell esds are taken into account individually in the estimation of esds in distances, angles and torsion angles; correlations between esds in cell parameters are only used when they are defined by crystal symmetry. An approximate (isotropic) treatment of cell esds is used for estimating esds involving l.s. planes.
Refinement. Refinement of F^2^ against ALL reflections. The weighted R-factor wR and goodness of fit S are based on F^2^, conventional R-factors R are based on F, with F set to zero for negative F^2^. The threshold expression of F^2^ > 2sigma(F^2^) is used only for calculating R-factors(gt) etc. and is not relevant to the choice of reflections for refinement. R-factors based on F^2^ are statistically about twice as large as those based on F, and R- factors based on ALL data will be even larger.

### Fractional atomic coordinates and isotropic or equivalent isotropic displacement parameters (Å^2^)

**Table:**

	*x*	*y*	*z*	*U*_iso_*/*U*_eq_	
C1	0.54437 (16)	0.2010 (2)	0.06349 (7)	0.0163 (4)	
C2	0.64357 (16)	0.1415 (2)	0.03776 (7)	0.0169 (4)	
H2	0.6318	0.0727	0.0119	0.020*	
C3	0.76100 (16)	0.1855 (2)	0.05087 (7)	0.0158 (4)	
C4	0.77916 (16)	0.2916 (2)	0.08860 (7)	0.0162 (4)	
H4	0.8574	0.3207	0.0974	0.019*	
C5	0.67901 (17)	0.35318 (19)	0.11272 (7)	0.0168 (4)	
C6	0.56157 (16)	0.3076 (2)	0.10094 (7)	0.0169 (4)	
H6	0.4952	0.3480	0.1179	0.020*	
C7	0.86620 (16)	0.1109 (2)	0.02637 (7)	0.0161 (4)	
C8	0.70035 (16)	0.4673 (2)	0.15257 (7)	0.0165 (4)	
C9	0.32778 (16)	0.1744 (2)	0.08031 (7)	0.0176 (4)	
H9A	0.2559	0.1698	0.0585	0.021*	
H9B	0.3330	0.2712	0.0944	0.021*	
C10	0.31395 (16)	0.0687 (2)	0.12475 (7)	0.0169 (4)	
C11	0.41173 (17)	−0.0068 (2)	0.14518 (7)	0.0206 (4)	
H11A	0.4889	0.0072	0.1313	0.025*	
C12	0.39619 (17)	−0.1030 (2)	0.18599 (7)	0.0205 (4)	
H12	0.4629	−0.1514	0.1998	0.025*	
C13	0.28067 (17)	−0.1271 (2)	0.20631 (7)	0.0178 (4)	
C14	0.18210 (17)	−0.0509 (2)	0.18628 (7)	0.0188 (4)	
H14A	0.1048	−0.0654	0.2000	0.023*	
C15	0.19867 (16)	0.0462 (2)	0.14616 (7)	0.0169 (4)	
H15	0.1324	0.0972	0.1332	0.020*	
C16	0.25906 (17)	−0.2367 (2)	0.24740 (7)	0.0187 (4)	
O1	0.85402 (11)	0.02572 (14)	−0.00993 (5)	0.0198 (3)	
O2	0.97202 (12)	0.14066 (14)	0.04773 (5)	0.0195 (3)	
O3	0.80429 (11)	0.49920 (14)	0.16624 (5)	0.0214 (3)	
O4	0.60297 (11)	0.52892 (14)	0.17105 (5)	0.0209 (3)	
O5	0.43311 (11)	0.14724 (14)	0.04881 (5)	0.0183 (3)	
O6	0.35857 (12)	−0.28772 (15)	0.26911 (5)	0.0240 (3)	
O7	0.15576 (12)	−0.27618 (14)	0.25925 (5)	0.0235 (3)	
H4A	0.627 (3)	0.600 (4)	0.1980 (12)	0.084 (11)*	
H6A	0.339 (3)	−0.362 (3)	0.2920 (11)	0.065 (9)*	
H2A	1.032 (3)	0.079 (3)	0.0334 (11)	0.067 (9)*	

### Atomic displacement parameters (Å^2^)

**Table:**

	*U*^11^	*U*^22^	*U*^33^	*U*^12^	*U*^13^	*U*^23^
C1	0.0158 (9)	0.0172 (10)	0.0160 (9)	−0.0019 (8)	−0.0005 (7)	0.0044 (7)
C2	0.0213 (10)	0.0149 (10)	0.0145 (9)	−0.0002 (8)	0.0001 (8)	0.0011 (7)
C3	0.0175 (9)	0.0159 (9)	0.0141 (9)	0.0003 (7)	0.0005 (7)	0.0030 (7)
C4	0.0147 (9)	0.0166 (10)	0.0172 (9)	−0.0010 (8)	−0.0009 (7)	0.0021 (7)
C5	0.0212 (10)	0.0148 (9)	0.0144 (9)	0.0006 (8)	0.0009 (8)	0.0037 (7)
C6	0.0165 (9)	0.0174 (10)	0.0168 (9)	0.0012 (8)	0.0024 (7)	0.0022 (7)
C7	0.0182 (10)	0.0163 (10)	0.0138 (9)	−0.0012 (8)	0.0004 (7)	0.0030 (7)
C8	0.0163 (10)	0.0161 (10)	0.0172 (9)	−0.0005 (8)	0.0003 (8)	0.0017 (7)
C9	0.0134 (9)	0.0205 (10)	0.0191 (10)	−0.0011 (8)	0.0010 (8)	−0.0008 (8)
C10	0.0181 (9)	0.0160 (10)	0.0165 (10)	−0.0008 (7)	−0.0009 (7)	−0.0041 (7)
C11	0.0151 (9)	0.0234 (11)	0.0232 (11)	−0.0020 (8)	0.0015 (8)	0.0025 (8)
C12	0.0176 (10)	0.0216 (10)	0.0222 (10)	0.0009 (8)	−0.0014 (8)	0.0009 (8)
C13	0.0193 (10)	0.0171 (10)	0.0169 (10)	−0.0007 (8)	−0.0008 (8)	−0.0022 (7)
C14	0.0154 (9)	0.0215 (10)	0.0194 (10)	−0.0016 (8)	0.0014 (8)	−0.0029 (8)
C15	0.0154 (9)	0.0188 (10)	0.0166 (10)	0.0014 (8)	−0.0007 (7)	−0.0037 (8)
C16	0.0204 (10)	0.0183 (10)	0.0173 (10)	0.0000 (8)	−0.0003 (8)	−0.0020 (8)
O1	0.0183 (7)	0.0211 (7)	0.0199 (7)	0.0009 (6)	0.0004 (5)	−0.0040 (6)
O2	0.0157 (7)	0.0219 (8)	0.0208 (7)	0.0008 (6)	−0.0011 (6)	−0.0037 (6)
O3	0.0161 (7)	0.0241 (8)	0.0240 (8)	−0.0006 (6)	−0.0014 (6)	−0.0058 (6)
O4	0.0151 (7)	0.0231 (8)	0.0245 (8)	0.0009 (6)	0.0010 (6)	−0.0073 (6)
O5	0.0145 (7)	0.0217 (7)	0.0186 (7)	−0.0023 (5)	0.0009 (5)	−0.0023 (5)
O6	0.0233 (8)	0.0250 (8)	0.0238 (8)	−0.0012 (6)	−0.0035 (6)	0.0072 (6)
O7	0.0204 (7)	0.0230 (7)	0.0270 (8)	−0.0007 (6)	0.0057 (6)	0.0041 (6)

### Geometric parameters (Å, º)

**Table:**

C1—O5	1.374 (2)	C9—H9A	0.9700
C1—C2	1.389 (3)	C9—H9B	0.9700
C1—C6	1.392 (3)	C10—C11	1.386 (3)
C2—C3	1.396 (3)	C10—C15	1.397 (3)
C2—H2	0.9300	C11—C12	1.387 (3)
C3—C4	1.395 (3)	C11—H11A	0.9300
C3—C7	1.488 (3)	C12—C13	1.391 (3)
C4—C5	1.387 (3)	C12—H12	0.9300
C4—H4	0.9300	C13—C14	1.393 (3)
C5—C6	1.392 (3)	C13—C16	1.484 (3)
C5—C8	1.490 (3)	C14—C15	1.380 (3)
C6—H6	0.9300	C14—H14A	0.9300
C7—O1	1.229 (2)	C15—H15	0.9300
C7—O2	1.316 (2)	C16—O7	1.232 (2)
C8—O3	1.232 (2)	C16—O6	1.316 (2)
C8—O4	1.303 (2)	O2—H2A	0.95 (3)
C9—O5	1.435 (2)	O4—H4A	0.99 (3)
C9—C10	1.512 (3)	O6—H6A	0.93 (3)
			
O5—C1—C2	115.15 (16)	C10—C9—H9B	109.0
O5—C1—C6	124.63 (16)	H9A—C9—H9B	107.8
C2—C1—C6	120.22 (17)	C11—C10—C15	118.67 (17)
C1—C2—C3	119.74 (17)	C11—C10—C9	122.34 (16)
C1—C2—H2	120.1	C15—C10—C9	118.99 (16)
C3—C2—H2	120.1	C10—C11—C12	121.00 (17)
C4—C3—C2	120.40 (17)	C10—C11—H11A	119.5
C4—C3—C7	120.68 (16)	C12—C11—H11A	119.5
C2—C3—C7	118.81 (16)	C11—C12—C13	119.93 (17)
C5—C4—C3	119.12 (17)	C11—C12—H12	120.0
C5—C4—H4	120.4	C13—C12—H12	120.0
C3—C4—H4	120.4	C12—C13—C14	119.39 (17)
C4—C5—C6	120.98 (17)	C12—C13—C16	121.51 (17)
C4—C5—C8	118.28 (16)	C14—C13—C16	119.05 (17)
C6—C5—C8	120.73 (16)	C15—C14—C13	120.27 (17)
C1—C6—C5	119.47 (17)	C15—C14—H14A	119.9
C1—C6—H6	120.3	C13—C14—H14A	119.9
C5—C6—H6	120.3	C14—C15—C10	120.70 (17)
O1—C7—O2	123.17 (17)	C14—C15—H15	119.6
O1—C7—C3	122.32 (16)	C10—C15—H15	119.6
O2—C7—C3	114.48 (15)	O7—C16—O6	123.74 (17)
O3—C8—O4	123.62 (17)	O7—C16—C13	121.80 (17)
O3—C8—C5	120.81 (16)	O6—C16—C13	114.45 (16)
O4—C8—C5	115.58 (15)	C7—O2—H2A	109.3 (17)
O5—C9—C10	113.10 (15)	C8—O4—H4A	109.0 (18)
O5—C9—H9A	109.0	C1—O5—C9	120.05 (14)
C10—C9—H9A	109.0	C16—O6—H6A	109.8 (17)
O5—C9—H9B	109.0		
			
O5—C1—C2—C3	−178.10 (15)	O5—C9—C10—C11	23.7 (2)
C6—C1—C2—C3	2.2 (3)	O5—C9—C10—C15	−156.45 (15)
C1—C2—C3—C4	−1.8 (3)	C15—C10—C11—C12	0.1 (3)
C1—C2—C3—C7	174.41 (16)	C9—C10—C11—C12	179.95 (17)
C2—C3—C4—C5	−0.3 (3)	C10—C11—C12—C13	1.4 (3)
C7—C3—C4—C5	−176.39 (16)	C11—C12—C13—C14	−1.8 (3)
C3—C4—C5—C6	1.9 (3)	C11—C12—C13—C16	175.62 (17)
C3—C4—C5—C8	−179.46 (16)	C12—C13—C14—C15	0.9 (3)
O5—C1—C6—C5	179.77 (16)	C16—C13—C14—C15	−176.60 (16)
C2—C1—C6—C5	−0.6 (3)	C13—C14—C15—C10	0.5 (3)
C4—C5—C6—C1	−1.5 (3)	C11—C10—C15—C14	−1.0 (3)
C8—C5—C6—C1	179.90 (16)	C9—C10—C15—C14	179.12 (17)
C4—C3—C7—O1	−173.84 (17)	C12—C13—C16—O7	−167.37 (18)
C2—C3—C7—O1	10.0 (3)	C14—C13—C16—O7	10.1 (3)
C4—C3—C7—O2	8.0 (2)	C12—C13—C16—O6	11.9 (3)
C2—C3—C7—O2	−168.23 (16)	C14—C13—C16—O6	−170.61 (16)
C4—C5—C8—O3	−4.8 (3)	C2—C1—O5—C9	165.51 (15)
C6—C5—C8—O3	173.81 (17)	C6—C1—O5—C9	−14.8 (3)
C4—C5—C8—O4	174.98 (16)	C10—C9—O5—C1	−84.35 (19)
C6—C5—C8—O4	−6.4 (3)		

### Hydrogen-bond geometry (Å, º)

*Cg*2 is the centroid of the C10–C15 ring.

**Table:**

*D*—H···*A*	*D*—H	H···*A*	*D*···*A*	*D*—H···*A*
O2—H2*A*···O1^i^	0.95 (3)	1.70 (3)	2.6426 (19)	176 (2)
O4—H4*A*···O7^ii^	0.99 (3)	1.62 (3)	2.6086 (19)	175 (3)
O6—H6*A*···O3^iii^	0.93 (3)	1.72 (3)	2.6486 (19)	179 (4)
C9—H9*A*···O1^iv^	0.97	2.51	3.272 (2)	135
C11—H11*A*···O3^v^	0.93	2.45	3.170 (2)	135
C14—H14*A*···O4^vi^	0.93	2.56	3.245 (2)	131
C9—H9*B*···*Cg*2^vii^	0.97	2.97	3.779 (2)	141

Symmetry codes: (i) −*x*+2, −*y*, −*z*; (ii) *x*+1/2, *y*+1, −*z*+1/2; (iii) *x*−1/2, *y*−1, −*z*+1/2; (iv) −*x*+1, −*y*, −*z*; (v) −*x*+3/2, *y*−1/2, *z*; (vi) −*x*+1/2, *y*−1/2, *z*; (vii) −*x*+1/2, *y*+1/2, *z*.
